# The Buffalo Academy of Medicine

**Published:** 1892-04

**Authors:** 


BUFFALO MEDICAL AND SURGICAL JOURNAL
A MONTHLY REVIEW OF MEDICINE AND SURGERY.

*EDITORS:*
THOMAS LOTHROP, M. D. - - WM. WARREN POTTER, M. D.

All communications, whether of a literary or business character, should be addressed to the managing editor: 284 Franklin Street, Buffalo, N. Y.

Vol. XXXI. APRIL, 1892. No. 9.

### THE BUFFALO ACADEMY OF MEDICINE.

There is a movement on foot to concentrate the medical intellect of the city in an organization with the above title. Last Autumn, the Buffalo Medical and Surgical Association, the Buffalo Obstetrical Society, the Buffalo Pathological Society, and the Buffalo Clinical Society, each appointed a committee from its members to meet the committee of the other societies to consider the advisability of the formation of such an organization. These committees met, and after full discussion, concluded that such action was advisable, and that the time was ripe for the formation of an academy. After making such report to their respective societies, the committees were continued in existence and directed to complete plans for such organization.

This work the committees have done. Reports of the plan of organization have been made to the Pathological Society and to the Obstetrical Society, and will be made, at their next meetings, to the Medical and Surgical Association and to the Clinical Society.

The Pathological and Obstetrical Societies, after full discussion of the plans as suggested by the committee, have voted to enter into the organization, if certain minor alterations be made in the plan.

In the proposed constitution, which is modeled after that of the New York Academy of Medicine, the object of the academy is stated to be "the promotion of the science and art of medicine, including the maintenance of a medical library and a museum."

It is expected that the academy will rent for a while and eventuually own a building which will be a center where the medical men of the city may meet, possess a library which all can consult, and a museum where any can study whatever particular subject he wishes to investigate.

It is to be hoped that the medical men of the city will enter into this undertaking in a liberal and generous spirit, and establish an academy worthy of a city which maintains two medical schools of such high standing, and numbers among its citizens medical men of national—nay, even world-wide—reputation as authorities in their profession.

The JOURNAL pledges itself to a hearty coöperation with the projectors of this commendable enterprise in perfecting it and promoting its greatest usefulness.

